# Avoid or Embrace? Practice Effects in Alzheimer’s Disease Prevention Trials

**DOI:** 10.3389/fnagi.2022.883131

**Published:** 2022-06-16

**Authors:** Andrew J. Aschenbrenner, Jason Hassenstab, Guoqiao Wang, Yan Li, Chengjie Xiong, Eric McDade, David B. Clifford, Stephen Salloway, Martin Farlow, Roy Yaari, Eden Y. J. Cheng, Karen C. Holdridge, Catherine J. Mummery, Colin L. Masters, Ging-Yuek Hsiung, Ghulam Surti, Gregory S. Day, Sandra Weintraub, Lawrence S. Honig, James E. Galvin, John M. Ringman, William S. Brooks, Nick C. Fox, Peter J. Snyder, Kazushi Suzuki, Hiroyuki Shimada, Susanne Gräber, Randall J. Bateman

**Affiliations:** ^1^Washington University in St. Louis School of Medicine, St. Louis, MO, United States; ^2^Warren Alpert Medical School of Brown University, Providence, RI, United States; ^3^Indiana University School of Medicine, Indianapolis, IN, United States; ^4^Eli Lilly and Company, Indianapolis, IN, United States; ^5^University College London, London, United Kingdom; ^6^University of Melbourne, Melbourne, VIC, Australia; ^7^The University of British Columbia, Vancouver, BC, Canada; ^8^The University of Rhode Island, Kingston, RI, United States; ^9^Mayo Clinic, Jacksonville, FL, United States; ^10^Feiniberg School of Medicine, Northwestern University, Chicago, IL, United States; ^11^Columbia University Irving Medical Center, New York, NY, United States; ^12^Miller School of Medicine, University of Miami, Miami, FL, United States; ^13^University of Southern California, Los Angeles, CA, United States; ^14^Neuroscience Research Australia, University of New South Wales Medicine, Randwick, NSW, Australia; ^15^Dementia Research Center, University College London, London, United Kingdom; ^16^The University of Tokyo, Tokyo, Japan; ^17^Osaka City University, Osaka, Japan; ^18^German Center for Neurodegenerative Disease (DZNE), Tübingen, Germany

**Keywords:** practice effects, Alzheimer’s disease, clinical trials, learning, assessment frequency, alternative forms

## Abstract

Demonstrating a slowing in the rate of cognitive decline is a common outcome measure in clinical trials in Alzheimer’s disease (AD). Selection of cognitive endpoints typically includes modeling candidate outcome measures in the many, richly phenotyped observational cohort studies available. An important part of choosing cognitive endpoints is a consideration of improvements in performance due to repeated cognitive testing (termed “practice effects”). As primary and secondary AD prevention trials are comprised predominantly of cognitively unimpaired participants, practice effects may be substantial and may have considerable impact on detecting cognitive change. The extent to which practice effects in AD prevention trials are similar to those from observational studies and how these potential differences impact trials is unknown. In the current study, we analyzed data from the recently completed DIAN-TU-001 clinical trial (TU) and the associated DIAN-Observational (OBS) study. Results indicated that asymptomatic mutation carriers in the TU exhibited persistent practice effects on several key outcomes spanning the entire trial duration. Critically, these practice related improvements were larger on certain tests in the TU relative to matched participants from the OBS study. Our results suggest that the magnitude of practice effects may not be captured by modeling potential endpoints in observational studies where assessments are typically less frequent and drug expectancy effects are absent. Using alternate instrument forms (represented in our study by computerized tasks) may partly mitigate practice effects in clinical trials but incorporating practice effects as outcomes may also be viable. Thus, investigators must carefully consider practice effects (either by minimizing them or modeling them directly) when designing cognitive endpoint AD prevention trials by utilizing trial data with similar assessment frequencies.

## Introduction

Phase 3 secondary prevention clinical trials in Alzheimer’s disease aim to demonstrate the efficacy of drug or other interventions in preserving or improving cognitive function in at-risk individuals. Such trials typically use the slowing of the rate of cognitive decline between a treatment arm and a placebo group as their primary efficacy endpoint (Sperling et al., 2014; Bateman et al., 2017; Cummings et al., 2020). Comprehensive neuropsychological test batteries are administered at regular intervals (e.g., every 6–12 months) to best characterize cognitive change across the course of the trial and to monitor for adverse events such as unexpected drops in performance. However, these repeated administrations may have unanticipated consequences for trial outcomes. Specifically, it is well-known that healthy adults typically improve in performance (termed “practice effects” or “PEs”) with repeated cognitive testing (Calamia et al., 2012). These PEs can be attributed to several factors including increased familiarity with task procedures, development of testing strategies, or memorization of specific stimuli. These gains are not limited to short time intervals and can persist for as long as 7 years (Salthouse et al., 2004) after just one exposure, a longer time span than a typical AD prevention trial. It is also important to consider that in symptomatic AD populations, where active neurodegenerative processes drive worsening cognitive performance, practice effects do not always translate to better performance from visit to visit. Rather, the competing forces of disease and PEs can manifest as attenuations of decline such that PEs may be observable as flat or simply less negative slopes.

For these reasons, potential PEs must be taken into consideration when planning a clinical trial. The two primary analytical models used in AD trials either analyze change from baseline to final test (e.g., mixed models for repeated measures or MMRM) or conceptualize change as linear from baseline to end of study (random intercept and slope models). When PEs are present but unaccounted for in statistical models, the magnitude of decline over the course of the trial can be drastically underestimated (Hassenstab et al., 2015; Jacobs et al., 2017) reducing the power to detect a treatment effect. Therefore, it may be desirable to minimize the influence of PEs in a clinical trial. One way to do so would be to include multiple “screening” sessions (Goldberg et al., 2015) which give participants experience with the cognitive battery prior to the initiation of treatment, as PEs tend to be largest after the first or second retest (Collie et al., 2003; Bartels et al., 2010). Other methods for minimizing PEs include the use of alternate forms, although this presents the additional challenge of verifying that the different forms are truly psychometrically equivalent (Gross et al., 2012), and yet still limit PEs due to familiarity. Computerized cognitive assessments, depending on the test paradigms, can protect against form-related PEs by randomly selecting stimuli for each test administration, creating an essentially endless number of alternate forms. But of course, this requires additional equipment and study management that can be costly and may not suit all trial protocols. Importantly, none of these approaches are entirely successful at eliminating practice effects (Beglinger et al., 2005; Falleti et al., 2006). Given the difficulties with eliminating PEs in cognitive studies, some studies have turned away from efforts at avoiding PEs opting instead to determine if incorporating PEs as outcomes themselves may reveal meaningful information about cognitive status. For example, several studies have shown that the attenuation of PEs in clinically healthy older adults can predict important outcomes such as biomarker status or risk of progression to symptomatic AD (Duff et al., 2011; Hassenstab et al., 2015; Machulda et al., 2017; Oltra-Cucarella et al., 2018; Samaroo et al., 2020). PEs may therefore serve as a subtle marker of early disease even if average cognitive trajectories are relatively flat. It is critical, therefore, to have a comprehensive understanding of factors that produce or exaggerate practice effects and to develop statistical tools to appropriately model them. Ultimately, the magnitude of PEs may serve as an alternative or supplementary endpoint for trials.

Similar to clinical trials, observational studies of AD provide systematic and longitudinal assessment of clinical, cognitive and pathological progression of the disease, albeit in the absence of a specific intervention. Although PEs have been relatively well studied in community-based observational studies of sporadic AD, to date, we are unaware of any systematic evaluation of PEs in the context of a clinical trial. One might expect that PEs would be attenuated in clinical trials if the study protocol includes a comprehensive screening assessment, which may provide exposure to the testing materials (Goldberg et al., 2015). Alternatively, in some cases, trial participants might be recruited from ongoing observational studies and hence are already familiar with the process of cognitive testing and may have exposure to the same test materials. Another important difference from observational studies is the role of participant expectations in clinical trials. Trial participants may exhibit enhanced PEs due to a type of placebo effect, wherein motivation and engagement may be higher in the trial compared to observational studies where expectations and motivations for participation may be different. As many trials rely on data from observational studies to select appropriate cognitive measures as endpoints and conduct power analyzes to determine the requisite sample sizes needed to detect a hypothetical treatment effect, it is critical to test the assumption that participants in observational studies will perform similarly to those engaged in clinical trial research. If these two populations differ in terms of PEs or overall cognitive trajectories, pre-specified cognitive endpoints selected based on observational study data may not be suitable for a clinical trial and sample sizes may be underestimated, among other concerns.

To address these issues, we present analyzes from the recently completed DIAN-TU 001 (TU) clinical trial (Mills et al., 2013) and the associated DIAN Observational study (OBS, Bateman et al., 2012). The DIAN-TU is a phase 2/3, double blind, placebo controlled study of disease modifying therapies in autosomal dominant AD (ADAD), a rare form of AD due to specific genetic mutations that has similar pathological and clinical presentations, other than in age at onset, as sporadic AD (Bateman et al., 2011). These genetic mutations cause AD with virtually 100% penetrance and onset of clinical symptoms begin at a predictable and typically much younger age than sporadic AD (Ryman et al., 2014). The expected number of years to symptom onset (EYO) can be calculated based on the participant’s age and the historical average age-at-symptomatic onset of gene-carriers with the same mutation or from the same family. The predictability of expected symptom onset as well as pathological similarities to the more common sporadic form of AD, makes ADAD a critical population in which to understand and build a model of cognitive, clinical, and pathological disease progression (McDade et al., 2018). To maintain participant blinding to their mutation status, ADAD mutation carriers (MCs) and non-carriers (NMCs) were enrolled in the trial, with all NMCs being assigned to placebo in a double blinded manner. The DIAN Observational study was launched in 2008 to provide natural history data on the progression of clinical, cognitive, and pathological changes in this population. Several participants who enrolled in the OBS study later enrolled in the TU study. We utilized the data from these two studies to answer the following questions: (1) Do PEs in ADAD vary as a function of mutation status or clinical status? (2) Do alternate forms that vary the stimuli across repeated administration (computerized battery vs. pen and paper) moderate the size of PEs? and (3) Do cognitive trajectories in clinical trials differ from those in observational only studies?

## Materials and Methods

A total of 384 participants were included in our analyzes. One-hundred ninety-three participants from the TU cohort and 191 from the OBS cohort. Both studies recruited a population of ADAD mutation carriers and non-carriers to determine the natural history (OBS) and to implement safe, efficient, and effective clinical trials that have the highest likelihood of success in advancing overall treatment (TU). Although the TU study was not powered to determine cognitive effects at the higher treatment doses that were ultimately used (5% power to detect a 30% slowing in the rate of cognitive decline), we have previously shown the absence of a treatment effect on cognitive outcomes in the TU (Salloway et al., 2021). Thus, given the relatively small group differences between treatment and placebo arms, for the present analyzes, all participants were combined and treatment arm [e.g., drug (solanezumab/gantenerumab) vs. placebo] was not considered. A small number of NMCs had clinical evidence for impairment (3 in the TU and 7 in OBS), these participants were removed prior to analysis due to small sample size, leaving a total of 374 participants available for analysis.

### Clinical/Cognitive Evaluation

Participants in both the TU and OBS studies underwent comprehensive clinical and cognitive evaluations. Presence and severity of dementia symptoms was ascertained using the Clinical Dementia Rating^®^ (CDR) scale (Morris, 1993). A global rating of 0 on the CDR reflects no dementia, while scores of 0.5, 1, 2, and 3 reflect very mild, mild, moderate, and severe dementia, respectively. The Mini-Mental State Exam (Folstein et al., 1975) (MMSE) was also given as a measure of general cognitive function.

The cognitive batteries were largely similar across the two studies. Neuropsychological tests that were given in common across the two cohorts have been described elsewhere (Storandt et al., 2014) and include Wechsler Memory Scale-Revised Logical Memory Immediate and Delayed Recall (Wechsler, 1987) and Digit Span, Trail making Parts A and B (Armitage, 1945), Category Fluency for Animals and Vegetables (Goodglass and Kaplan, 1983), and Digit Symbol Substitution from the Wechsler Adult Intelligence Scale-Revised (Wechsler, 1981). In the TU, participants were also administered the Cogstate computerized battery which included Identification, Detection, One-Back, One Card Learning, and the International Shopping List test. These measures have been described extensively elsewhere (Hammers et al., 2011; Lim et al., 2012). In the TU, most of these tests were administered every 6 months except for category fluency and the MMSE which were measured annually. All tests utilized the same versions at each testing occasion with the exception of the Cogstate tests which produced randomly generated stimuli at each occasion. Assessment frequency in the OBS study ranged from every 1–3 years depending on clinical status and when the participant entered the study. The OBS study has enrolled over 575 participants to date, but for the purpose of these analyzes, we selected participants that matched the enrollment criterion for the TU. We included as many participants as possible who met the following criteria: baseline global CDR score of 1 or less and estimated years to EYO range from –15 to +10 years (Salloway et al., 2021; See Table 1 for full demographics). For the purposes of these analyzes, participants who were initially enrolled in the OBS study and then transitioned to the TU (41% of the TU CDR 0 carriers, 32% of the TU CDR > 0 carriers and 33% of the TU non-carriers started in the OBS study) were included in the TU cohort but were excluded from analyzes in the OBS cohort.

**TABLE 1:** Demographic characteristics of the clinical trial (TU) and observational (OBS) study cohorts.

	DIAN-TU			DIAN Obs		
	NMC	MC CDR 0	MC CDR > 0	NMC	MC CDR 0	MC CDR > 0
*N*	46	85	59	115	35	34
Age	42.0 (9.2)	40.9 (8.5)	49.2 (10.1)	41.3 (8.9)	38.7 (9.5)	46.0 (8.3)
EYO	–4.5 (6.3)	–5.8 (6.3)	2.7 (4.8)	–6.1 (6.8)	–8.3 (6.0)	1.2 (3.9)
Sex (% female)	20 (43%)	45 (53%)	28 (47%)	70 (61%)	25 (71%)	22 (65%)
Education	15.5 (3.2)	15.6 (3.2)	14.1 (2.6)	14.9 (2.8)	14.3 (2.7)	13.2 (3.2)
Number of assessments	7.3 (3.6)	9.5 (2.2)	8.1 (2.5)	2.2 (1.3)	2.7 (1.1)	3.3 (1.2)
Length of follow-up	3.1 (1.8)	4.2 (1.1)	3.6 (1.3)	2.5 (2.5)	3.6 (1.9)	2.9 (1.6)

*Results are reported as mean (SD) where appropriate.*

### Statistical Analysis

Our analyzes proceeded in several steps. We first compared cognitive trajectories in the TU battery between NMCs, CDR 0 MCs and CDR > 0 MCs. We constructed linear mixed effects (LME) models for each cognitive test and predicted cognition from baseline EYO, time-in-study (hereafter referred to as “time”), group and the group by time interaction. A random intercept and random slope of time was also included in all models with an unstructured covariance matrix. Follow-up contrasts were constructed to compare slopes on each test between the NMCs and the CDR 0 MCs, and between the CDR 0 MCs and the CDR > 0 MCs. For ease of comparison across tests, all outcomes were z-scored to the baseline mean and standard deviation of the CDR 0 non-carriers so that a score of “0” represents the score of a relatively cognitively normal participant. Scores were oriented such that a positive slope indicates an improvement over time and a negative slope indicates decline.

A second set of LMEs were constructed to compare performance in the TU vs. the OBS study. Specifically, we analyzed performance on each cognitive test as a function of time, group (NMC, CDR 0 MC, and CDR > 0 MCs) and cohort (TU vs. OBS), and included all of the two and three-way interactions while also controlling for baseline EYO. All models were fit in the R statistical computing software (version 4.0.5, R Core Team, 2021) using the lme4 package (version 1.1.27.1, Bates et al., 2015). *P*-values were obtained using the lmerTest (version 3.1.3, Kuznetsova et al., 2017) package. To ensure that no influential, outlying data points were unduly biasing our results, we used the infleunce.ME package (Nieuwenhuis et al., 2012) to iteratively remove a single participant from each model and re-run the statistical analysis. We checked for a change in statistical significance in key model parameters (specifically, the group by time or group by cohort by time interactions) when a given participant was removed. Across all the analyses we conducted, none of those parameters changed significance suggesting no single person was exerting undue influence on these results. Finally, although a relatively large set of statistical comparisons were conducted in order to fully describe practice effects across a range of cognitive tests, no corrections for multiple comparisons were made.

## Results

### Analysis 1: Clinical Trial Only

**FIGURE 1:**
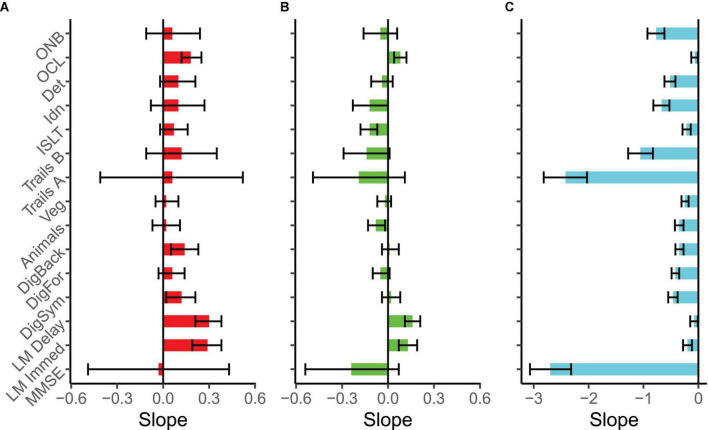
Slope estimates and 95% confidence intervals for each test and clinical group in the Dominantly Inherited Alzheimer Network Trials Unit (DIAN-TU). Slopes can be considered significant if the CI does not encompass zero. All tests were scaled such that a negative slope indicates decline. Slopes are in z-score units change per year. **(A)** Plots non-carriers (*N* = 46), **(B)** plots Clinical Dementia Rating (CDR) 0 mutation carriers (*N* = 85), and **(C)** plots CDR > 0 mutation carriers (*N* = 59). Due to dramatic performance differences across groups, the X-axis scale is not identical across the panels. ONB, one-back; OCL, one card learning; Det, detection; Idn, identification; ISLT, international shopping list; Veg, category fluency for vegetables; animals, category fluency for animals; DigFor and DigBack, digit span forward and backward; LM, logical memory; MMSE, Mini Mental State Exam.

Slopes over time for each cognitive test and each group are illustrated in Figure 1. Intercepts and slope scores for each test can also be found in Supplementary Table 1. Not surprisingly, the MC CDR > 0 group evinced significant decline on all cognitive measures with some of the largest effects occurring on tests of perceptual speed and attention (Cogstate Detection, Identification and One back, Digit Symbol Substitution and Trail Making Part A). In contrast, cognitive trajectories for the MC CDR 0 group were relatively flat with a few notable exceptions. There was significant decline on Category Fluency for Animals, the ISLT and the Identification test, suggesting that measures of semantic fluency, episodic memory and attention are sensitive to preclinical cognitive decline. Interestingly, the Logical Memory immediate and delayed recall tests showed significant improvement over time in this population as did Cogstate One Card Learning, a test of visual learning ability. NMCs did not decline on any measure, which was expected in a relatively young and cognitively healthy cohort. Showing the classic pattern of practice effects, NMCs exhibited significant improvement over time compared to a zero slope on several measures including Logical Memory Immediate and Delayed Recall, Digit Symbol Substitution, Digit Span Backward and One Card Learning.

**FIGURE 2:**
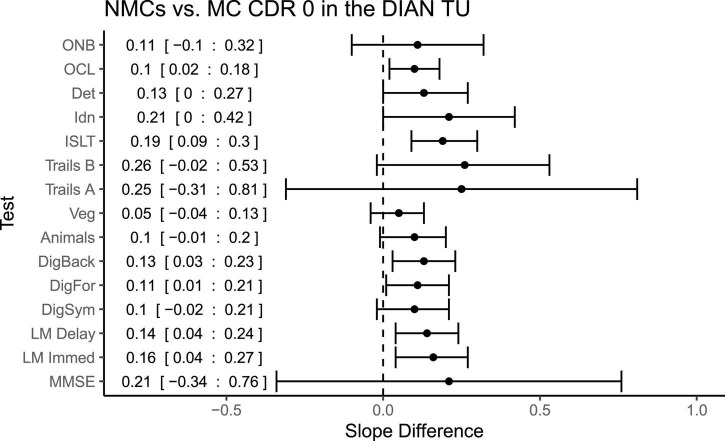
Differences in slopes (and 95% CIs) for each test between non-carriers and CDR 0 mutation carriers in the clinical trial (TU). A positive slope difference indicates a larger or more positive slope (improvement) in the non-carriers compared to mutation carriers. Mean difference and 95% confidence intervals are pasted along the left side.

In order to determine disease effects on learning and decline, we next compared slopes between the NMCs and the MC CDR 0 group (shown in Figure 2) to determine if differences in rate of change distinguished the groups. Slopes (reflecting change per year in z-score units) were significantly different between these two groups on the following measures: One Card Learning (Difference = 0.10, *p* = 0.01, *CI* = 0.02:0.18), Logical Memory Immediate (Difference = 0.16, *p* = 0.009, *CI* = 0.04:0.27), Logical Memory Delayed (Difference = 0.14, *p* = 0.007, *CI* = 0.04:0.24), Digit Span Forward (Difference = 0.11, *p* = 0.04, *CI* = 0.006:0.21), Digit Span Backward (Difference = 0.13, *p* = 0.02, *CI* = 0.03:0.23) and the ISLT (Difference = 0.19, *p* < 0.001, *CI* = 0.08:0.30). These results indicate that while both MCs and NMCs exhibited PEs (see Figure 1) on the Logical Memory and One Card Learning tests, practice-related gains were significantly larger in the NMCs. Moreover, NMCs improved over time on the Digit Span Backward test whereas the MCs showed no significant change. Finally, the NMCs did not show improvement or decline on ISLT whereas the MCs significantly declined.

### Analysis 2: Observational Versus Clinical Trial

**FIGURE 3:**
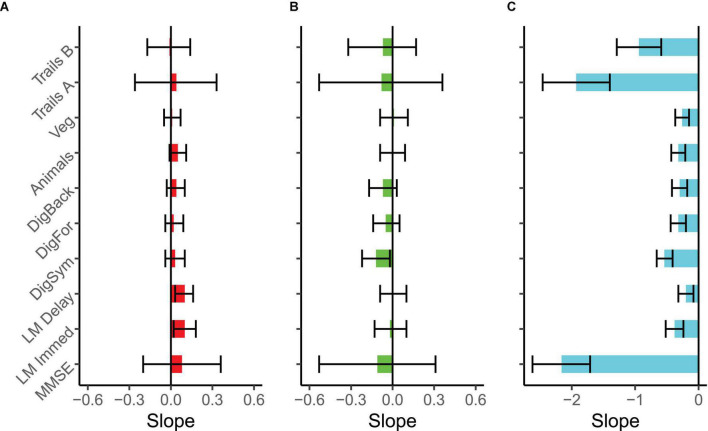
Slope estimates and 95% confidence intervals for each test and clinical group in the DIAN-Observational (DIAN-Obs) study. Slopes are expressed as z-score units change per year and can be considered significant if the CI does not encompass zero. All tests were scaled such that a negative slope indicates decline. **(A)** Plots non-carriers (*N* = 115), **(B)** plots CDR 0 mutation carriers (*N* = 35), and **(C)** plots CDR > 0 mutation carriers (*N* = 34). Due to dramatic performance differences across groups, the X-axis scale is not identical across the panels.

Intercepts and slopes for the eligible participants in the OBS study are provided in Supplementary Table 2, and slopes for each test and group are plotted in Figure 3, showing time-dependent changes. First, similar to the TU, the MC CDR > 0 group in the observational study declined significantly on all measures. Second, the MC CDR 0 group again showed relatively flat cognitive trajectories with the notable exception of the Digit Symbol Substitution test which significantly declined by 0.12 z-score units per year. Most importantly, there was no hint of practice related improvements in the MC CDR 0s, with lack of evidence of positive slope estimates, on any of the cognitive measures. Finally, the NMC group significantly improved on the Logical Memory Immediate and Delayed Recall tests but the slopes for the other measures were relatively flat and not significantly different from zero.

**FIGURE 4:**
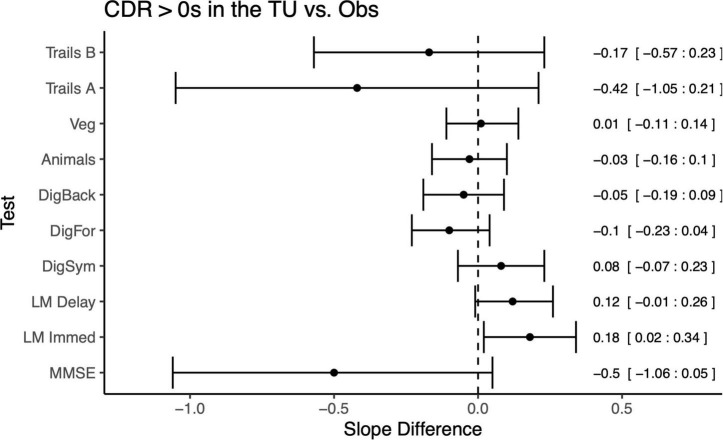
Differences in slopes (and 95% CIs) for each test between CDR > 0 mutation carriers in TU vs. the Obs study. A positive slope difference indicates a larger or more positive slope (improvement) in the TU compared to the Obs study. Some tests, such as Logical Memory, had more improvement or practice effects in the TU vs. Obs. Mean differences and 95% CIs presented along the right side of the graph.

**FIGURE 5:**
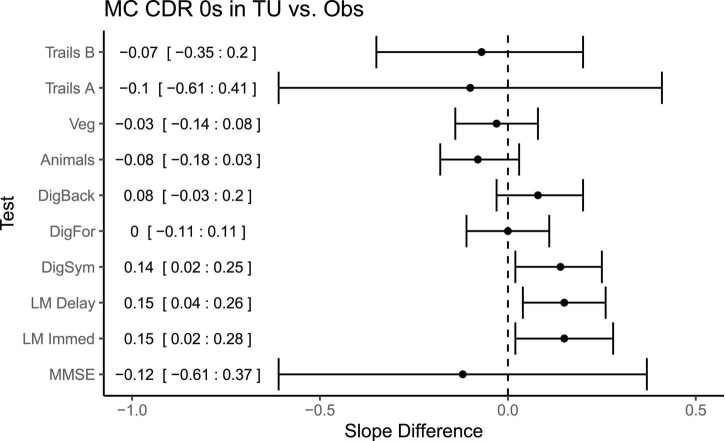
Differences in slopes (and 95% CIs) for each test between CDR 0 mutation carriers in TU vs. the Obs study. A positive slope difference indicates a larger or more positive slope (improvement) in the TU compared to the Obs study. Some tests, such as Logical Memory and Digit Symbol, had more improvement or practice effects in the TU vs. Obs. Mean differences and 95% CIs presented along the left side of the graph.

**FIGURE 6:**
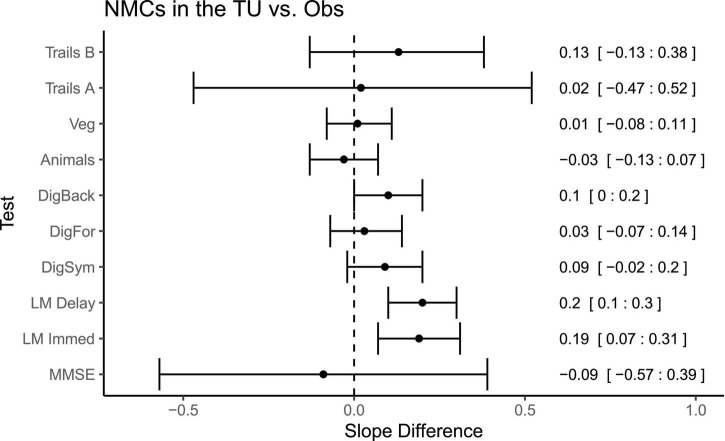
Differences in slopes (and 95% CIs) for each test between non-mutation carriers in TU vs. the Obs study. A positive slope difference indicates a larger or more positive slope (improvement) in the TU compared to the Obs study. Some tests, such as Logical Memory had more improvement or practice effects in the TU vs. Obs. Mean differences and 95% CIs presented along the right side of the graph.

Direct comparisons between the symptomatic MCs in the OBS and TU cohorts (Figure 4), revealed no significant differences in slopes between the cohorts on any measure with the exception of Logical Memory Immediate Recall (Difference = 0.18, *p* = 0.03, *CI* = 0.02:0.34), in which participants in the TU showed slightly less decline than in OBS. Interestingly, a number of differences emerged when comparing the asymptomatic MCs across TU and OBS (Figure 5). Specifically, on the Digit Symbol Substitution test (Difference = 0.14, *p* = 0.02, *CI* = 0.02:0.25), Logical Memory Immediate (Difference = 0.15, *p* = 0.03, *CI* = 0.02:0.28) and Delayed recall (Difference = 0.15, *p* = 0.007, *CI* = 0.04:0.26) slopes were markedly less negative in the TU as compared to the OBS study. Finally, in the comparison of NMCs (Figure 6), the OBS participants improved less on Logical Memory Immediate (Difference = 0.19, *p* = 0.003, *CI* = 0.07:0.31) and Delayed recall (Difference = 0.20, *p* < 0.001, *CI* = 0.10:0.30) compared to the TU participants.

## Discussion

In this study, we compared performance on a comprehensive cognitive battery in two cohorts to answer several important questions regarding practice related improvements in observational studies and clinical trials in AD populations.

### Question 1: Does Mutation Status or Clinical Status Moderate Practice Effects in the Dominantly Inherited Alzheimer Network Trials Unit?

Clinical status was an important predictor of PEs in the DIAN-TU. Specifically, individuals who were CDR > 0 at entry significantly declined on all cognitive measures and therefore did not show practice-related gains. This is not to say that PEs were not present in this group, only that any gains associated with practice were overshadowed by the decline attributable to AD pathology. More importantly, mutation status in the CDR 0 groups also predicted magnitude of change in the TU. MC CDR 0s declined significantly over time on measures of attention, episodic memory, and semantic fluency whereas NMCs showed no change in these domains. Interestingly, differences in performance between MC CDR 0s and NMCs also emerged on the ISLT (list recall, MCs declined more than NMCs), Logical Memory (narrative recall, MCs improved less than NMCs), Digit Span (working memory, MCs improved less than NMCs), and One Card Learning (visual learning, MCs improved less than NMCs). Together these findings suggest that differences in the magnitude of practice related improvements in domains of memory and learning might serve as a sensitive and supplemental indicator of preclinical AD.

### Question 2: Do Alternative Forms Influence Practice Effects?

We expected *a priori* that computerized measures from the Cogstate battery might show less practice effects due to the nature of randomized stimuli which generates essentially unlimited alternate forms. For example, many of these tasks use playing cards as stimuli presented in a newly randomized order at each administration. Such a design reduces the possibility of memorizing specific items which can be a contributor to PEs. This contrasts with Logical Memory in the DIAN studies, for example, which presents the same narrative each time the test is taken.

Our hypothesis was largely supported. Most of the computerized tests were resistant to practice effects in the NMCs or sensitive to decline in the MCs (e.g., ISLT and Identification tests). Practice related gains were apparent on the One Card Learning test and due to the nature of the randomized stimuli, it is assumed that participants are developing or learning some strategy besides rote memorization to improve over time. One possibility is that this test might be particularly amenable to visual strategies such as the method of loci (Gross et al., 2014). As the cards are shown one at a time, participants may over time learn to organize the items in a meaningful fashion (e.g., into poker hands or by suit) which might aid recall.

### Question 3: Are Practice Effects Similar Across Clinical Trials and Observational Studies?

One of the most important questions addressed in this study was whether cognitive trajectories were similar across a clinical trial cohort and an observational study. For participants who were CDR > 0 at baseline, the answer was clearly “yes”. Regardless of the cohort, MC CDR > 0s declined significantly over time and the magnitude of change did not differ significantly between the TU and OBS with the sole exception of Logical Memory Immediate Recall. This may reflect disease progression such that symptomatic MCs have declined to the extent that any practice related gains were outweighed by the task demands. An interesting but complex question for future studies is to determine the point at which PEs are effectively overwhelmed by disease related declines.

For the MC CDR 0s, however, a few critical differences did emerge. Specifically, OBS participants declined at a faster rate than the TU participants on the Digit Symbol Substitution test and improved less on the Logical Memory Immediate and Delayed Recall tests. One obvious possible explanation for these differences is the assessment frequency across the two studies (every 6 months in the TU, ∼ every 2 years in OBS). This explanation is likely for the Logical Memory tests, where participants will hear the same story at each testing occasion which reinforces encoding and aids in recall. It is less clear why Digit Symbol Substitution would show such enhanced practice effects in the TU when other measures of speed and executive function did not (e.g., the Trail Making tests). Studies of retest have shown performance gains on Digit Symbol Substitution, but this test typically demonstrates less gains than episodic memory measures (Calamia et al., 2012). Thus, frequency of assessment needs to be carefully considered during trial design.

Another important possibility is an enhanced placebo effect in the DIAN-TU. Specifically, TU participants were randomized to treatment vs. placebo at a ratio of 3:1. Thus, there may have been a greater expectation of being on active drug which may have then impacted cognitive performance. Regardless of the underlying mechanisms, these differences in practice related gains are particularly noteworthy as the Logical Memory and Digit Symbol tests feature heavily in multiple cognitive composite endpoints (Sperling et al., 2014; Bateman et al., 2017). Investigators should keep in mind potential differences between observational and trial cohorts when planning their studies and conducting power analyzes.

Using alternate instrument forms has been shown in some studies to be a viable strategy to reduce PEs. For example, a meta-analysis of test/retest effects found substantial reductions in performance gains when alternate forms were used for verbal list learning measures (Calamia et al., 2012). This finding is similar to the results shown here, in which the computerized tests were largely resistant to practice-related gains. The one exception we found was One Card Learning, a visual learning test that uses randomly generated sequences of cards such that there are essentially hundreds of alternate forms. This task produced the largest PEs in asymptomatic MCs enrolled in the DIAN-TU clinical trial. We could not, however, determine if this was due solely to clinical trial participation, as this measure was not collected with sufficient samples in the OBS study for comparison. In a recent study, the developers of the One Card Learning test made a shorter and less difficult version of the test (as evidenced by less floor effects in symptomatic AD participants) that demonstrated no PEs in young cognitively normal participants across very short retest intervals (White et al., 2021). The authors argue that the increased difficulty and length of the longer version of the task may lead to participants forming strategies that in turn lead to more PEs.

Although our results indicate that rates of change on key cognitive outcomes may be underestimated in clinical trials due to the presence of these practice effects, it is important to highlight situations in which these practice effects might limit the ability to detect treatment effects. Specifically, in clinical trials that include a placebo arm in which participants undergo identical clinical and cognitive assessments as participants on active treatment, the negative impact of practice effects may be minimal, to the extent that practice effects manifest similarly in placebo vs. treated patients. However, this also assumes that the influence of improved cognition due to treatment is additive, rather than interactive, with improved cognition due to practice effects, which may not be the case. Moreover, the primary cognitive outcome is often a composite score formed of multiple tests. If some tests exhibit practice effects while others do not, as is the case in the present study, decline on a global composite score may be very small, limiting the power to detect any differences among groups.

It is unclear if attempts to avoid or reduce practice effects are futile. Completely avoiding practice effects does seem an impossible task. One of the most fundamental aspects of human behavior is adaptation, or learning. As we and others have previously shown, in the context of a cognitive assessment this learning is not just limited to familiarity with test materials but also to process factors like test strategies, effort, demand characteristics, and expectancy effects, among others (Beglinger et al., 2005; Hassenstab et al., 2015; Machulda et al., 2017). Instead of avoiding PEs, trials that enroll cognitively normal or mildly affected participants might consider designs and statistical models that anticipate and account for the influence of PEs. Such protocols might include extended baseline designs that allow cluster assessments prior to dosing in so-called “run-in” designs (Frost et al., 2008). Less emphasis might be placed on spreading assessments out at regular time intervals (e.g., one assessment every 6 months) in favor of clustering assessments at key read-out times and averaging across the clusters, which might not only minimize the effects of practice but also reduce individual variability in scores (Valdes et al., 2016). An alternative strategy is to incorporate PEs as outcomes themselves. Several recent studies have deliberately measured learning effects in cognitively normal older adults at risk for AD (Hassenstab et al., 2015; Baker et al., 2020; Lim et al., 2020; Samaroo et al., 2020). Effect sizes differentiating participants with biomarker-confirmed preclinical AD from those with normal biomarker levels are extraordinarily large for these paradigms, suggesting that PEs may be a highly sensitive indicator of disease progression.

There are many strengths to this study including use of a comprehensive cognitive battery on very well-characterized clinical cohorts, designed comparability between an observational study and clinical trial, enrolling the same population for both studies, and frequent assessments over many years. However, some limitations need to be noted. First, because this is a study of ADAD, a very rare form of AD, the sample sizes included here could be considered small. Moreover, it is unclear whether differences in practice related gains will translate to the more common sporadic form of the disease. Second, some participants in these studies may become aware of their mutation status and this might alter their cognitive outcomes (Aschenbrenner et al., 2020). It is unknown whether the number of participants who did and did not learn their status were similar across the two studies. Third, we did not have data from the Cogstate testing battery in the DIAN-OBS study which precluded a comparison of PEs between the trial and observational study on these measures. Finally, we conducted many statistical tests due to the large cognitive battery that was administered and although many effects could have been predicted *a priori* this could be seen as an additional limitation.

Nevertheless, these results highlight three important points. (1) Practice effects were highly evident in the DIAN-TU-001 clinical trial in asymptomatic mutation carriers and non-carriers. (2) Alternate forms may have attenuated practice effects, but not for all measures. (3) The magnitudes of practice effects were larger in the DIAN-TU-001 clinical trial than seen in a well-matched sample from the DIAN Observational study, suggesting that more frequent assessments and placebo effects in clinical trials may drive increases in practice effects. Clinical trials that utilize a cognitive endpoint should carefully consider the potential for practice effects and select statistical modeling strategies that can incorporate them directly.

## Data Availability Statement

The datasets presented in this article are not readily available because risk of identifying individual participants and/or risk to ongoing trial activities. Requests to access the datasets should be directed to https://dian.wustl.edu/our-research/for-investigators/diantu-investigator-resources.

## Ethics Statement

The studies involving human participants were reviewed and approved by Local Ethics Committees at TU sites. The patients/participants provided their written informed consent to participate in this study.

## Dominantly Inherited Alzheimer Network Trials Unit (DIAN-TU)

Data used in the preparation of this article were obtained from the Dominantly Inherited Alzheimer Network Trials Unit (DIAN-TU). As such, the study team members within the DIAN-TU contributed to the design and implementation of DIAN-TU and/or provided data but may not have participated in the analysis or writing of this report. A complete listing of the DIAN-TU Study Team Members can be found at dian.wustl.edu, DIAN-TU Study Team.

## Author Contributions

AA statistical analysis, development of study hypotheses, and initial draft of the manuscript. JH development of study hypotheses, acquisition of study data, and revised manuscript for intellectual content. GW, YL, CX, EC, and KH revised manuscript for intellectual content. EM, DC, SS, MF, RY, CJM, CLM, G-YH, GS, GD, SW, LH, JG, JR, WB, NF, PS, KS, HS, SG, and RB acquisition of study data and revised manuscript for intellectual content. All authors contributed to the article and approved the submitted version.

## Author Disclaimer

The content is solely the responsibility of the authors and does not necessarily represent the official views of the National Institutes of Health.

## Conflict of Interest

EC, KH, and RY are full-time employees and/or stockholders of Eli Lilly. RB has received funding from Avid Radiopharmaceuticals, Jansse, Hoffman La-Roche/Genentech, Eli LIlly & Co., Eisai, Biogen, AbbVie, and Bristol Meyer Squibbs, has royalties/licenses from C2N Diagnostics, consulting fees from Eisai, Amgen, and Hoffman La-Roche, honoraria from the Korean Dementia Association and the American Neurological Association, and is on the data safety monitoring board or advisory board for Roche/Genentech and Biogen. Unrelated to this article, RB serves as the principal investigator of the DIAN-TU, which is supported by the Alzheimer’s Association, GHR Foundation, an anonymous organization, and the DIAN-TU Pharma Consortium (Active: Eli Lilly and Company/Avid Radiopharmaceuticals, F. Hoffman-La Roche/Genentech, Biogen, Eisai, and Janssen. Previous: Abbvie, Amgen, AstraZeneca, Forum, Mithridion, Novartis, Pfizer, Sanofi, and United Neuroscience). In addition, in-kind support has been received from CogState and Signant Health. G-YH has received research support as a clinical trials site investigator from Anavax, Biogen, Eli Lilly, and Roche, has received research grants from the CIHR, Alzheimer Society of Canada, and NIA/NIH, is supported by the Ralph Fisher Professorship in dementia research from the Alzheimer Society of British Columbia, and has participated in expert advisory committees sponsored by Biogen and Roche. GD’s research is supported by NIH (K23AG064029), the Alzheimer’s Association, and Chan Zuckerberg Initiative, and he serves as a consultant for Parabon Nanolabs Inc., as a Topic Editor (Dementia) for DynaMed (EBSCO), and as the Clinical Director of the Anti-NMDA Receptor Encephalitis Foundation (Inc, Canada; uncompensated) and owns stock in ANI pharmaceuticals. DH, former Department Head of Neurology where the research was conducted, is an inventor on patents for one of the treatments (solanezumab), which has been tested in the DIAN-TU clinical trials. If solanezumab is approved as a treatment for Alzheimer’s disease or Dominantly Inherited Alzheimer’s Disease, Washington University, and will receive part of the net sales of solanezumab from Eli Lilly, which has licensed the patents related to solanezumab from Washington University. The remaining authors declare that the research was conducted in the absence of any commercial or financial relationships that could be construed as a potential conflict of interest.

## Publisher’s Note

All claims expressed in this article are solely those of the authors and do not necessarily represent those of their affiliated organizations, or those of the publisher, the editors and the reviewers. Any product that may be evaluated in this article, or claim that may be made by its manufacturer, is not guaranteed or endorsed by the publisher.
