# Supplementary material for: Avoid or Embrace? Practice Effects in Alzheimer’s Disease Prevention Trials
Source: Front Aging Neurosci. 2022 Jun 16;14:883131. doi: 10.3389/fnagi.2022.883131 (PMC9244171; doi:10.3389/fnagi.2022.883131)
Supplement: Supplementary file 1 [file Table_1.DOCX]

Note: Veg = Category Fluency for Vegetables, Animals = Category Fluency for Animals., DigFor and DigBack = Digit Span Forward and Backwards, LM = Logical Memory, MMSE = Mini Mental State Exam.
